# Genomic structure and selection history across Angus populations worldwide: insights from ROH, selection mapping, and functional analyses

**DOI:** 10.1007/s00335-025-10188-y

**Published:** 2025-12-22

**Authors:** Henrique A. Mulim, Gabriel S. Campos, Fernando F. Cardoso, Victor Breno Pedrosa, Kajal Latimer, Lindsay R. Upperman, A. J. Knowles, Andre Garcia, Kelli Retallick, Steve Miller, Hinayah Rojas de Oliveira

**Affiliations:** 1 Department of Animal Science, Purdue University, West Lafayette, IN 47907 USA; 2 Department of Animal Biosciences, Interbull Centre, S75007 Uppsala, Sweden; 3 Brazilian Agricultural Research Corporation, Embrapa South Livestock Center, Bage, RS Brazil; 4 Neogen Corporation, Lincoln, NE 68504 USA; 5 Canadian Angus Association, Rocky View County, AB T4A 0E2 Canada; 6 Red Angus Association of America, Commerce City, CO 80022 USA; 7 Angus Genetics Inc. American Angus Association, Saint Joseph, MO 64506 USA; 8 AGBU, a Joint Venture of New South Wales Department of Primary Industries and Regional Development and the University of New England, Armidale, NSW 2351 Australia

## Abstract

Angus cattle, originally from Scotland, have been selectively bred for over 400 years, making them one of the most prominent beef breeds globally. Known for their adaptability, natural polled traits, and high-quality beef, Angus cattle have been intensively selected for growth, body size, and feed efficiency. This study investigates the genetic diversity, selection history, and key genomic regions across five Angus populations from the USA, Canada, Australia, Brazil, and Red Angus of America. Genomic data from 71,283 animals born between 1961 and 2024 were analyzed using Principal Component Analysis (PCA), phylogenetic tree construction, and Runs of Homozygosity (ROH), with the Generation Proxy Selection Mapping (GPSM) approach used to assess selection history. Functional annotation identified candidate genes and pathways related to selection. Our analysis revealed both similarities and differences across populations. The PCA and FST metrics showed minimal differentiation between the American, Canadian, Australian, and Brazilian populations, with greater differentiation observed in the Red Angus population. The ROH analysis revealed that the Brazilian population had the highest number of ROHs. The ROH islands identified on BTA8 and BTA13 in the American and Australian populations were linked to traits like body weight, marbling, and tenderness. The GPSM identified significant markers associated with body weight and growth in all populations, reflecting ongoing selection pressures. This study highlights the potential of genomics to improve our understanding of Angus cattle’s genetic architecture and selection history. It underscores the feasibility of integrating global populations for more accurate genomic evaluations, enhancing genetic predictions, and supporting sustainable beef production worldwide.

**Supplementary Information:**

The online version contains supplementary material available at 10.1007/s00335-025-10188-y.

## Introduction

Angus cattle, a taurine breed originating from Scotland, have been selectively bred for over 400 years (Nawaz et al. 2024; Vasconcellos et al. 2003). Known for its adaptability to various climates and environments, the breed gained popularity due to its naturally polled (hornless) characteristic and high-quality beef (Smakuyev et al. 2021). Comparative evaluations through the US MARC Germplasm Evaluation Program have shown that Angus has undergone major shifts in growth and carcass traits relative to other breeds over the past decades (Hruska et al. 2001; Langlie et al. 2019; Engle et al. 2024). During the twentieth century, intensive selection focused on key traits, such as growth rate, body size, and feed efficiency, significantly enhancing the breed’s productivity (Campos et al. 2014). This selective breeding effort, in parts facilitated by branded beef programs, established Angus as one of the most prominent breeds globally, highly valued for its marbled meat and efficient feed-to-meat conversion (Nawaz et al. 2024). Nowadays, Angus cattle play a vital role in the beef industry, especially in countries such as the United States, Canada, Brazil, and Australia, where advanced genetic and genomic selection methods continue to improve their performance in both feedlot and pasture systems.

Angus cattle were first introduced to Australia in the 1820s, with the first documented import occurring in 1840 when eight animals were brought to Tasmania (Angus Australia 2024). The breed quickly became popular among Australian cattle producers due to its high-quality beef and adaptability to Australia’s diverse climates. To promote and register the breed in the country, the Angus Australia Association was established in 1946. Structured genetic evaluation in Angus cattle began in the 1970s with the establishment of the Angus Herd Improvement Scheme and the National Beef Recording Scheme, leading to the Angus Herd Improvement Register in 1977 and the Group TACE system in the early 1980s, which enabled across-herd genetic comparisons (Angus Australia 2024). The Angus Sire Benchmarking Program, initiated in 2009, created a large reference population with genotypic and phenotypic data. Genomic information was officially incorporated into evaluations in 2011, and a single-step model integrating pedigree, performance, and genomic data was implemented in 2017 to enhance breeding value accuracy (Angus Australia 2024).

In the United States, Angus cattle were introduced in 1873, when George Grant imported four Angus bulls from Scotland to Kansas (American Angus Association, 2024a). Grant crossbred these bulls with Texas Longhorn cows in the United States, resulting in hardy, hornless black calves that thrived on winter rangelands. Valued for their high-quality beef, strong maternal abilities, and climate adaptability, Angus cattle quickly gained popularity in the United States (Smakuyev et al. 2021). In 1883, the American Angus Association was established to promote and standardize the breed, maintaining its official registry. The first official genetic evaluation in the American Angus Association was performed in 1974 (Retallick et al. 2022). Later in 2009, the first genomic-enhanced expected progeny difference (GE-EPDs) were launched, marking the first use of genomic selection in the US beef industry (Retallick et al. 2022). Initially, a multi-step method was used to perform the evaluations, but in 2017, the single-step genomic was introduced in the American Angus evaluation (Retallick et al. 2022).

The history of Angus cattle in Canada is similar to their introduction and growth in the United States, with some specific regional distinctions. For instance, the first documented importation into Canada occurred in 1860, when a few animals were brought from Scotland to Quebec (Canadian Angus Association 2024a). In 1905, the Canadian Angus Association was established to support the breed’s development and maintain a national registry of Angus animals. In Canada, The first genetic evaluation was conducted through the Government of Canada's Record of Performance Program in 1994. The Association later joined the American Angus and Red Angus Associations in their genetic evaluations. In 2018, the Canadian Angus Association implemented a combined genetic and genomic evaluation for both its Black and Red Angus population (Canadian Angus Association 2018).

In Brazil, Angus cattle were first imported in 1906 from Uruguay, specifically to the Rio Grande do Sul state (Brazilian Angus Association 2024). The Brazilian Angus Association was officially established in 1963, supporting breed improvement through initiatives such as the exchange of genetic material between countries and the use of advanced genomic biotechnologies. Although Brazil maintains an independent breed association and breeding program, imported genetic material continues to strengthen genetic links between Brazilian and global Angus populations (Cardoso et al. 2020; ASBIA 2024). The genetic evaluation of Angus cattle in Brazil began in 1974 with the Promebo® breeding program, focused on improving growth, carcass quality, and later, adaptability and tick resistance (Campos et al. 2022). Traditional evaluations based on pedigree and phenotypes were initially used. With advancements in genomics, Brazil adopted single-step genomic BLUP (ssGBLUP) to integrate pedigree, phenotypic, and genomic data. The first genomic evaluation for Brazilian Angus cattle was reported by Campos et al. (2022).

The Red Angus breed shares its origins with the Aberdeen Angus, tracing back to Scotland (Nawaz et al. 2024; Vasconcellos et al. 2003). It is suggested that the red color originated in the eighteenth century when English Longhorn cattle, predominantly red, were crossbred with black polled cattle to improve size and strength. While the resulting offspring were typically black, they carried the recessive red gene (Red Angus Association of America 2009). In the United States, both black and red Angus cattle were initially registered together under the American Aberdeen Angus Association. However, in 1917, the association restricted registration to black Angus only to preserve a pure black lineage. As red calves became less common in American herds, seven breeders founded the Red Angus Association of America in 1945, in order to promote and register the red breed (Red Angus Association of America 2009). In 1995, the Red Angus Association of America adopted Total Herd Reporting (THR), for improve the completeness and accuracy of performance records used in the estimation of expected progeny differences (Red Angus Association of America 2009). Genomic-enhanced EPDs were incorporated beginning in the early 2010s, when high-density SNP genotyping and single-step evaluation methods were integrated into the national genetic evaluation system (Red Angus Association of America 2012, 2016). In contrast to the United States, where black and red Angus are registered separately, many major beef-producing countries, including Brazil and Canada, maintain a single herdbook for both color variants (Cardoso et al. 2020; Brazilian Angus Association 2024).

The use of genomic data to evaluate signs of selection in livestock animals can provide precise insights into the effects of natural and artificial selection on different populations (Marchesi et al. 2018). This data allows the identification of selection signatures and the reconstruction of population history with high resolution, offering insights that are not accessible through pedigree or phenotypic data alone. Therefore, the objectives of this study were to: (1) estimate population stratification across the American Angus (AAA), Canadian Angus (CAA), Australian Angus (AUS), American Red Angus (RAAA), and Brazilian Angus (BRA) populations; (2) quantify and classify the Runs of Homozygosity (ROH) in each population; (3) characterize genomic similarities and differences among these populations; (4) investigate the selection architecture to reconstruct the selection history of each population using the Generation Proxy Selection Mapping (GPSM) method; and (5) perform gene annotation and functional analyses to identify potential candidate genes and metabolic pathways associated with genomic regions located in ROH islands and under selection over time (GPSM). We expect that the findings from this study will contribute to advancing the integration of genomic selection and international genetic evaluations in Angus cattle. By revealing both shared and population-specific genomic regions under selection, our results offer valuable insights to improve the accuracy of genomic predictions, inform cross-country breeding strategies, and support the development of globally connected reference populations.

## Material and methods

### Genomic information

This study analyzed genomic data from five populations of Angus cattle, comprising a total of 71,091 animals born between 1961 and 2024. The Angus Society of Australia (Angus Australia) and the American Angus Association provided genomic data for 15,000 animals each. The Canadian Angus Association contributed genotypic data for 14,808 animals. In all three associations, the animals were genotyped using the BovineSNP50 BeadChip, which contains 54,609 SNP markers. The dataset provided by the Canadian Angus Association consisted of 11,727 Black Angus and 3,081 Red Angus animals. The animals were randomly selected by the respective associations, with the only criterion being to have a known year of birth. The genotypic data included animals born between 1992 and 2024 for the Angus Australia Association, 1969 and 2024 for the American Angus Association, and 1988 and 2024 for the Canadian Angus Association.

The Red Angus Association of America provided genomic information for 12,506 Red Angus (RAAA) animals born between 1961 and 2024. These animals were genotyped using six different SNP panels, with densities ranging from 7 to 105K. After combining the SNPs and performing quality control, the data were imputed to a density of 75,807 markers using the FImpute software version 3 (Sargolzaei et al. 2014). The Brazilian Angus Association (Promebo® 2020) provided genomic data for 13,777 Brazilian Angus (85% black and 15% red) animals born between 1988 and 2023. These animals were genotyped using fourteen different SNP panels, with densities ranging from 35 to 150K. After combining the SNP panels and performing quality control, the data were imputed to a final density of 78,837 SNP markers. Details regarding the imputation process performed in the Brazilian population are available in Campos et al. (2022). Genotypic information for all populations was updated to the ARS-UCD1.2 genome assembly (Rosen et al. 2020).

### Quality control

Genotypic quality control procedures were tailored to the specific analyses performed in this study. For instance, for the identification of ROH, only data with a call rate above 95% for both animals and genotypes were used. For the other analysis performed in this study, additional quality control criteria included minor allele frequency (MAF) higher than or equal to 0.05, and Hardy–Weinberg equilibrium (HWE) p-value ≥ 1 × 10^–6^. Moreover, SNPs located on non-autosomal chromosomes were removed from the analyses. The number of animals and markers removed by each quality control criteria, for each population, is shown in Table 1.

**Table 1:** Number of animals and SNP markers removed based on each quality control criterion, and summary of the final genomic data from American (AAA), Australian (AUS), Canadian (CAA), Brazilian (BRA), and Red Angus (RAAA) populations

	AAA	AUS	CAA	BRA	RAAA
Animal call-rate	0	0	0	0	0
Genotype call-rate	1,418	500	33	0	0
non-autosomal	1,749	1,749	1,749	0	0
Minor Allele Frequency (MAF)	11,657	12,571	11,815	5,543	157
Hardy–Weinberg equilibrium	742	229	11,306	1,837	1,696
*Runs of homozygosity (ROH)*					
Total animals	15,000	15,000	14,808	13,777	12,506
Total markers	51,442	52,360	52,860	78,837	75,807
*Other analysis*					
Total animals	15,000	15,000	14,808	13,777	12,506
Total markers	39,043	39,560	29,706	71,457	73,954

### Principal component analysis

To evaluate the genetic similarities among populations, a principal component analysis (PCA) was performed using the PLINK software (Purcell et al. 2007). The PCA was based on the standardized variance of the genomic relationship matrix (**G**), where the covariance of each SNP was divided by its respective variance. Only SNPs common to all populations (after the complete quality control and linkage disequilibrium pruning (r^2^ > 0.8), i.e., 11,122 markers) were used. The number of markers in common between each population can be found in Supplementary Material 1. The genomic relationship matrix (**G**) was calculated according to the equation proposed by VanRaden (2008):

$$ {\varvec{G}} = \frac{{\left( {{\varvec{M}} - 2{\varvec{P}}} \right)\left( {{\varvec{M}} - 2{\varvec{P}}} \right)^{\prime } }}{{2\Sigma p_{i} \left( {1 - p_{i} } \right)}} $$

where **M** is a matrix of allele A counts, p_i_ is the allele A frequency for the ith SNP, and **P** is a matrix where each row contains the values of p_i_.

### Phylogenetic tree

The analyses were performed using the ape (Paradis et al. 2004), phangorn (Schliep 2011), and SNPRelate (Zheng et al. 2012) packages, all available in the R software (R Development Core Team 2009). Each package was used as appropriate for specific tasks, i.e.: ape and phangorn facilitated phylogenetic tree construction, while SNPRelate handled genomic data processing of SNP data stored in Genomic Data Structure (GDS) format. To enable efficient SNP data manipulation in R, PLINK binary files were first converted to GDS format using SNPRelate. Genotype data were then accessed from the GDS file, allowing calculation of mean SNP values per breed origin (country). For each SNP, the mean genotype value across all individuals from a given breed origin was calculated, resulting in a matrix of mean genotype values by breed origin. This matrix captured the genetic composition of each breed origin and served as the foundation for genetic similarity calculations. An identity-by-state (IBS) distance matrix was created using the breed origin-level genotype means to estimate genetic similarity between breed origins. A phylogenetic tree was subsequently generated from this distance matrix using the Neighbor-Joining (NJ) method (Saitou and Nei 1987), as implemented in the ape package. The NJ algorithm provides an unbiased representation of genetic relationships among the breed origins, based on shared genetic variants. Finally, phangorn was used to visualize and build the genetic clustering and relatedness within the Angus cattle populations.

### Proportion of polymorphic SNPs, heterozygosity, and average pairwise genetic distance

For each population, we determined the proportion of polymorphic SNPs after performing within-population quality control, focusing exclusively on SNPs with MAF greater than 5% (0.05). Observed heterozygosity (HO) was calculated for each individual within each population by dividing the count of heterozygous genotypes by the total number of genotypes. This observed heterozygosity was then compared to the expected heterozygosity (HE) under Hardy–Weinberg equilibrium. Both HO and HE values were calculated after the complete quality control was performed for each population, using the PLINK 1.9 software (Purcell et al. 2007).

To assess genetic differences between individuals within each population, we calculated the average pairwise genetic distance (D_ST_), defined as one minus the average proportion of shared alleles between two individuals. The D_ST_ metric was calculated using the following formula:

$$ D_{ST} = \frac{{IBS_{2} + \left( {0.5 \times IBS_{1} } \right)}}{m} $$

where IBS_1_ and IBS_2_ indicate the number of loci where one or both alleles, respectively, are identical-by-state (IBS), and m is the total number of loci. Linkage disequilibrium pruning was performed before calculating genetic distance, with a window size of 50 SNPs, shifting by 5 SNPs, and a variance inflation factor of 2. All these calculations were performed using the PLINK 1.9 software (Purcell et al. 2007).

### Linkage disequilibrium

Linkage disequilibrium (r^2^) was also estimated using the PLINK software (Purcell et al. 2007). The LD was calculated as the squared correlation between two alleles at different loci, i.e.

$$ LD = \frac{{D^{2} }}{f\left( A \right)f\left( a \right)f\left( B \right)f\left( b \right)} $$

where $D=f\left(AB\right)-f(A)f(B)$, and $f\left(AB\right),f\left(A\right),f\left(a\right),f\left(B\right), and f(b)$ are the observed frequencies of AB, A, a, B, and b, respectively. To assess the decay of r^2^ with increasing marker distance, a binning approach was used. The mean r^2^ was calculated for each distance from 10 to 100 kb in 10 kb intervals, and for distances beyond 100 kb, in 100 kb intervals up to 1,000 kb (1 Mb). During preliminary analysis (not shown), it was determined that each bin should include at least 50 paired SNP markers to provide a reliable estimate of mean r^2^. Linkage disequilibrium was estimated as the squared correlation between genotype allele counts at pairs of loci. The analyses were performed using the command: –r2 dprime-signed with-freqs –ld-window 100000 –ld-window-r2 0. This option also reports the signed D′ and allele frequencies, enabling a comprehensive assessment of LD patterns across the genome.

### Consistency of the gametic phase

Consistency of the gametic phase (CGP) was calculated by taking the square root of the r^2^ values and applying the sign of the linkage disequilibrium (D) measure. The formula used to calculate D is described as:

$$ D = p\left( {ab} \right) - p\left( a \right)p\left( b \right) $$

where p(a) is the frequency of allele a, p(b) is the frequency of allele b, and p(ab) is the frequency of the allele-pair carrying allele a at the first locus and allele b at the second locus. The CGP was estimated as the Pearson correlation coefficient of the signed square root of r^2^ values (√r^2^ × sign(D)), calculated for shared SNP pairs between populations. This transformation retains both the magnitude and direction of linkage disequilibrium, allowing the comparison of LD phase consistency across populations. To estimate CGP, only the SNPs that remained after the complete quality control and were shared between each population pair were used (N = 11,122). The same distance and binning approach described in the linkage disequilibrium section was used for the CGP.

### Detection of runs of homozygosity

Runs of homozygosity (ROH) were identified using the PLINK software (Purcell et al. 2007), following the parameters established by Mulim et al. (2022). The total number of markers used in each population is shown in Table 1. Specifically, ROHs were detected using sliding windows of 50 SNPs, with a minimum requirement of 30 consecutive SNPs, and a minimum length of 500 kb for a region to be classified as a ROH. The SNP density was set at a minimum of 1 SNP per 50 kb, and with a maximum allowed gap of 1,000 kb between consecutive SNPs. A window threshold of 0.05 was applied, permitting one heterozygous SNP and one missing SNP within the window. After detection, the ROHs were grouped into five categories based on their length: < 2 Mb, 2–4 Mb, 4–8 Mb, 8–16 Mb, and > 16 Mb. Markers identified within a ROH in at least 50% of the population were classified as ROH islands.

### Generation proxy selection mapping

The Generation Proxy Selection Mapping (GPSM) approach was applied to each population to detect alleles that have changed in frequency over time (Rowan et al. 2021). This method uses a genome-wide linear mixed model, with the individual’s generation number or a proxy (e.g., birth year) as the dependent variable. The following model was used to detect changes in allele frequency over time, for each population, using the GCTA software (Yang et al. 2011):

$$ {\varvec{y}} = \mu + {\varvec{X}}_{i} b_{i} + {\varvec{Zg}} + {\varvec{e}} $$

where **y** is a vector of individual generation proxies (i.e., birth year), µ is the sample mean, **X**_i_ is the vector of SNP genotypes for each individual at SNP i, *b*_i_ is the effect of SNP i, **g** is the vector of random polygenic effects ~ $N(0,{\varvec{G}}{\sigma }_{g}^{2})$, **Z** is the incidence matrix connecting **y** to the random polygenic effects in **g**, **e** is the vector of random residuals ~ $N(0,{\varvec{I}}{\sigma }_{e}^{2})$, **G** is the genomic relationship matrix (created following the first method shown in VanRaden 2008), ${\sigma }_{g}^{2}$ is the genomic variance, **I** is an identity matrix, and ${\sigma }_{e}^{2}$ is the residual variance. Supplementary Material 2 presents the distribution of genotypes by year in each population.

The GSPM analysis was performed using the *–mlma* flag available in the GCTA software. Quantile–quantile (Q-Q) plots were examined to assess the extent of false-positive signals. A Bonferroni multiple-test correction was used to set the significance threshold, with SNPs considered statistically significant if their p-values were below the 5% Bonferroni-corrected type-I error rate (i.e., α = 0.05/total number of SNPs after quality control for each population; Table 1).

### Investigation of genomic regions and functional analyses

Significant genomic regions identified through the GSPM analyses, along with SNP markers located within the ROH islands identified in this study, were selected for gene annotation and functional analysis. The GALLO package (Fonseca et al. 2020), available in the R software (R Development Core Team 2009), was used to perform gene and QTL annotation. Gene and QTL annotation were performed using the *Bos taurus taurus* data from the Ensembl database (version ARS-UCS1.2; Rosen et al. 2020), and the Animal QTL database (Hu et al. 2022), respectively. A genomic window of 100 Kb upstream and downstream of the markers was used for gene and QTL identification. The QTL enrichment analyses were performed using the *qtl_enrich* function in GALLO, which tests the overrepresentation of specific QTL categories across chromosomes using a hypergeometric test. The resulting p-values were adjusted for multiple testing using the false discovery rate (FDR) method, and QTL categories with an adjusted p < 0.05 were considered significantly enriched. Identified genes were subjected to functional analysis using the *gprofiler2* package (Peterson et al. 2020), also available in R. The enrichment was assessed across multiple functional databases (Gene Ontology: Biological Process, Cellular Component, and Molecular Function; KEGG pathways). Significance was determined using the FDR correction method, and terms with an adjusted p < 0.05 were considered significantly enriched.

## Results

### Population stratification

**Fig. 1:**
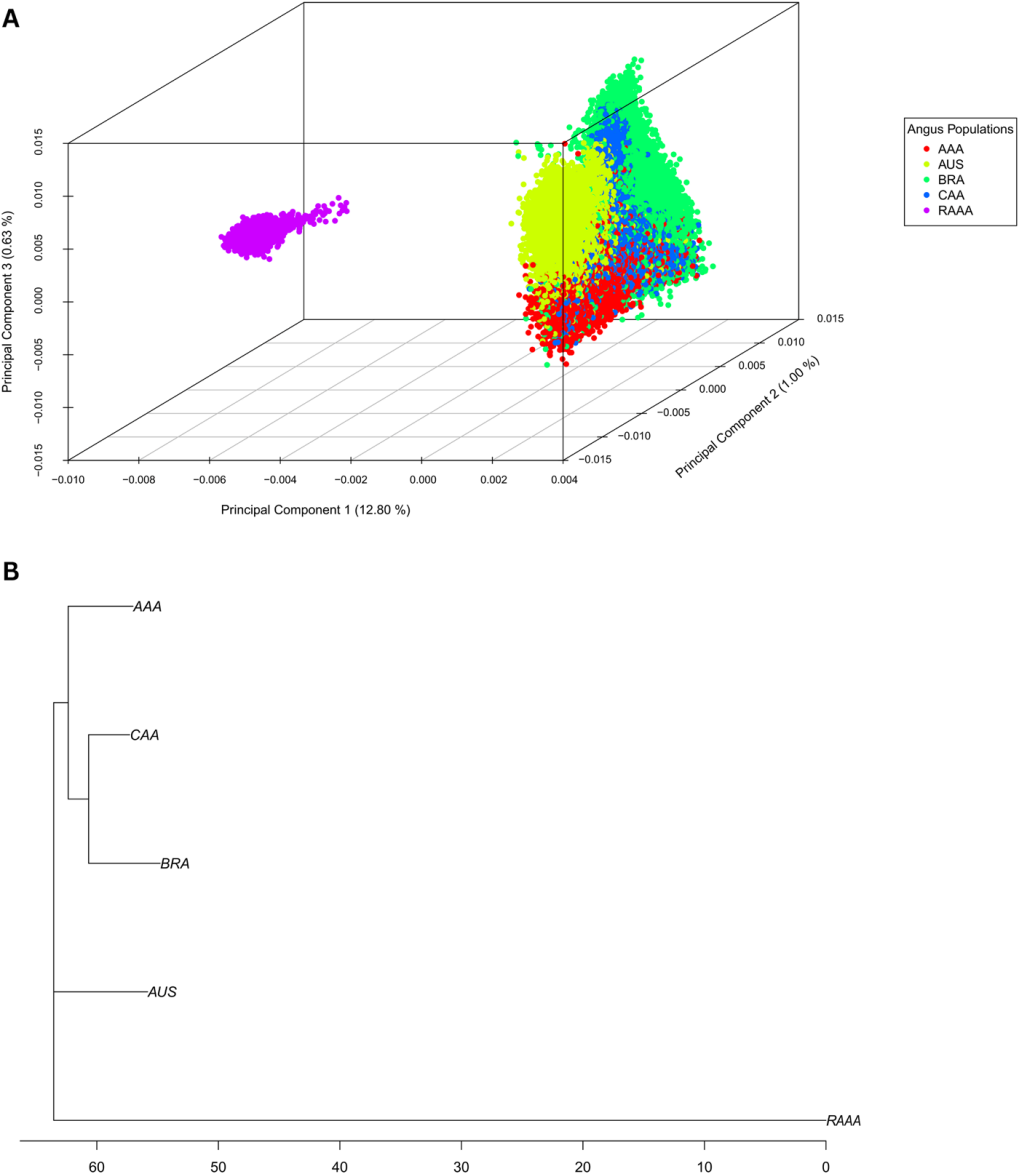
Principal component analysis (**A**) and phylogenetic tree (**B**) of American (AAA), Australian (AUS), Canadian (CAA), Brazilian (BRA), and Red Angus (RED) cattle populations. The X-axis represents genetic distance, expressed as the number of nucleotide substitutions per site. The scale bar indicates 0.05 substitutions per site

Figure 1 shows the population stratification of Angus based on the PCA (1a) and phylogenetic tree (1b). The first three principal components explained 14.43% of the variation among individuals, being the first PCA to explain more than 12% of the variation among populations, and the third to explain less than 1% of the variation. Two distinct clusters were identified: one grouping AAA, BRA, CAA, and AUS populations and another consisting exclusively of individuals from the RAAA population. This clustering pattern is supported by the phylogenetic tree, which shows the four populations being more closely related to each other than to the RAAA population.

Table 2 shows the genetic distance metrics within each population, along with average linkage disequilibrium, average marker distances, and proportion of polymorphic SNPs, as well as the expected and observed heterozygosity for each population.

**Table 2:** Genetic distance, average linkage disequilibrium (LD), average SNP distance (SNP Dist), proportion of polymorphic SNPs, and heterozygosity metrics and their respective standard deviation across American (AAA), Australian (AUS), Canadian (CAA), Brazilian (BRA), and Red Angus (RAAA) populations

	AAA	AUS	CAA	BRA	RAAA
Genetic distance	0.70 ± 0.01	0.70 ± 0.01	0.70 ± 0.01	0.68 ± 0.01	0.68 ± 0.01
LD	0.13 ± 0.17	0.13 ± 0.17	0.11 ± 0.16	0.11 ± 0.16	0.11 ± 0.16
SNP Dist (Mb)	0.5013 ± 0.2855	0.5014 ± 0.2855	0.5008 ± 0.2857	0.4982 ± 0.28821	0.4990 ± 0.28817
Polymorphic SNPS	0.787	0.770	0.784	0.930	0.998
Heterozygosity observed	0.37 ± 0.12	0.37 ± 0.12	0.37 ± 0.11	0.40 ± 0.10	0.40 ± 0.10
Heterozygosity expected	0.37 ± 0.12	0.37 ± 0.12	0.38 ± 0.12	0.41 ± 0.10	0.41 ± 0.10

In most cases, these metrics are similar across populations, with the primary difference being the proportion of polymorphic SNPs. Notably, AUS, AAA, and CAA have a lower proportion of polymorphic SNPs compared to BRA, and RAAA. The linkage disequilibrium decay for each population is shown in Supplementary Material 3.

Table 3 displays the average F_ST_ index for all populations studied. In summary, the average FST index ranged from 0.004 ± 0.006 for the AAA-CAA and CAA-BRA pairs to 0.152 ± 0.216 between AUS and RAAA.

**Table 3:** Pairwise average fixation index (FST) values with standard deviation for genetic differentiation among American (AAA), Australian (AUS), Canadian (CAA), Brazilian (BRA), and Red Angus (RAAA) populations

	CAA	AUS	BRA	RAAA
AAA	0.004 ± 0.006	0.008 ± 0.011	0.009 ± 0.012	0.151 ± 0.216
CAA		0.010 ± 0.013	0.004 ± 0.006	0.145 ± 0.216
AUS			0.015 ± 0.020	0.152 ± 0.216
BRA				0.145 ± 0.217

### Consistency of the gametic phase

**Fig. 2:**
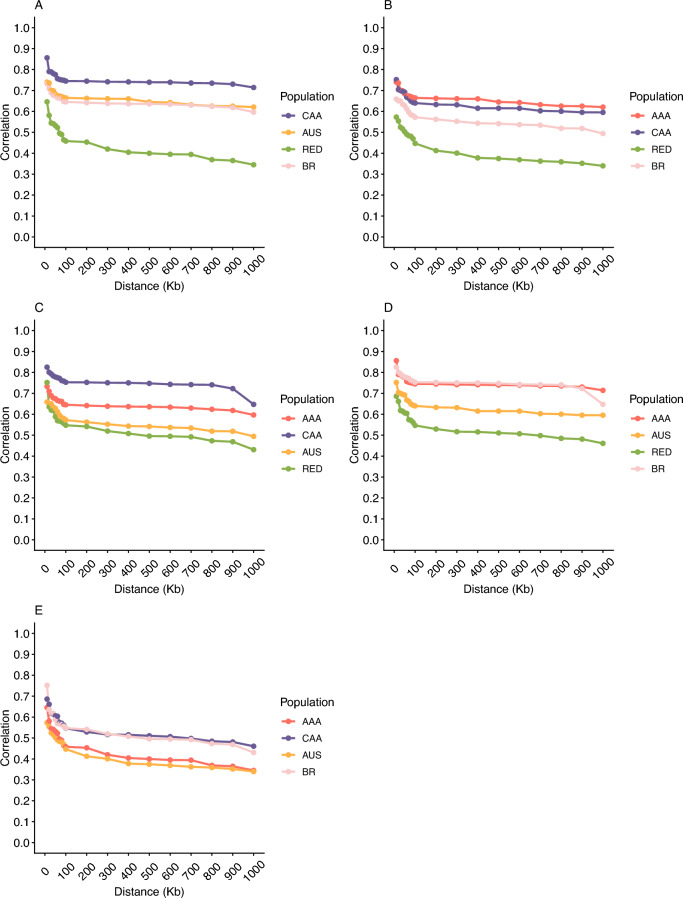
Consistency of gametic phase across distances and pairwise combinations in American (**A**, AAA), Australian (**B**, AUS), Brazilian (**C**, BRA), Canadian (**D**, CAA), and Red Angus (**E**, RAAA) cattle populations

Figure 2 shows the consistency of the gametic phase across all population pairs. Overall, the lowest correlation at the shortest distance (10 Kb) was observed between AUS and RAAA (0.67), while the highest correlation occurred between AAA and CAA (0.85 Fig. 2A, D). Populations AAA, CAA, and AUS exhibited consistently high correlations (> 0.60) across all evaluated distances. The RAAA population consistently showed the lowest correlations with all other populations across all distances. The BRA population demonstrated high correlation values with AAA and CAA at all distances, exceeding 0.70 in the shortest distance (Fig. 2A, C, D).

### Runs of homozygosity

**Fig. 3:**
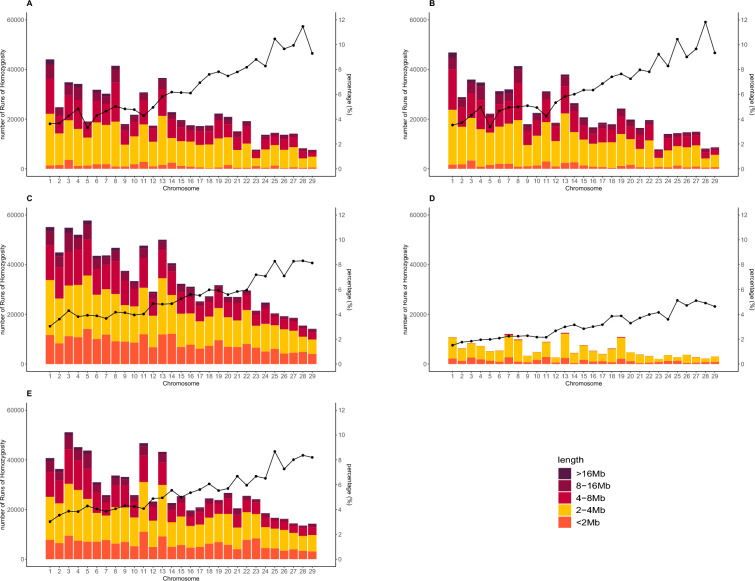
Chromosomal coverage and classification of runs of homozygosity in American (**A**), Australian (**B**), Brazilian (**C**), Canadian (**D**), and Red Angus (**E**) cattle populations. Percentages indicate the proportion of each chromosome’s physical length covered by ROHs in each population

Table 4 shows the minimum, maximum, and average numbers of runs of homozygosity (ROHs) per individual, along with the total and average lengths of ROHs across populations. Among the populations, BRA was the only one to exhibit at least one ROH in every individual, as well as the highest total and individual counts of ROHs. On average, CAA had the lowest number of ROHs per individual, with 11.27 ± 22.63 ROHs, while BRA had the highest average, with 37.85 ± 22.63 ROHs per individual. In terms of ROH length, AUS showed the highest average length of ROHs, measuring 5,125.70 ± 4,810.37 Kb. Figure 3 shows the classification of ROHs by length and chromosome.

**Table 4:** Summary of runs of homozygosity (ROH) metrics across American (AAA), Australian (AUS), Brazilian (BRA), and Red Angus (RAAA) populations

	AAA	AUS	BRA	CAA	RAAA
Min	0	0	1	0	0
Max	85	102	167	61	141
Mean	22.24 ± 13.11	23.18 ± 13.66	37.85 ± 22.63	11.27 ± 5.56	33.56 ± 19.87
Total	636,638	664,369	1,002,240	166,891	806,437
Avg length (kb)	5,091.53 ± 4,613.84	5,125.70 ± 4,810.37	4,323.06 ± 4,423.29	2,405.80 ± 573.11	4,345.93 ± 4,270.73

*Min* minimum number of ROHs identified per individual, *Max* maximum number of ROHs identified per individual, *Mean* average number of ROHs identified per individual, *Total* total number of ROHs identified in the population, *Avg length* average length of ROHs identified in the population

**Fig. 4:**
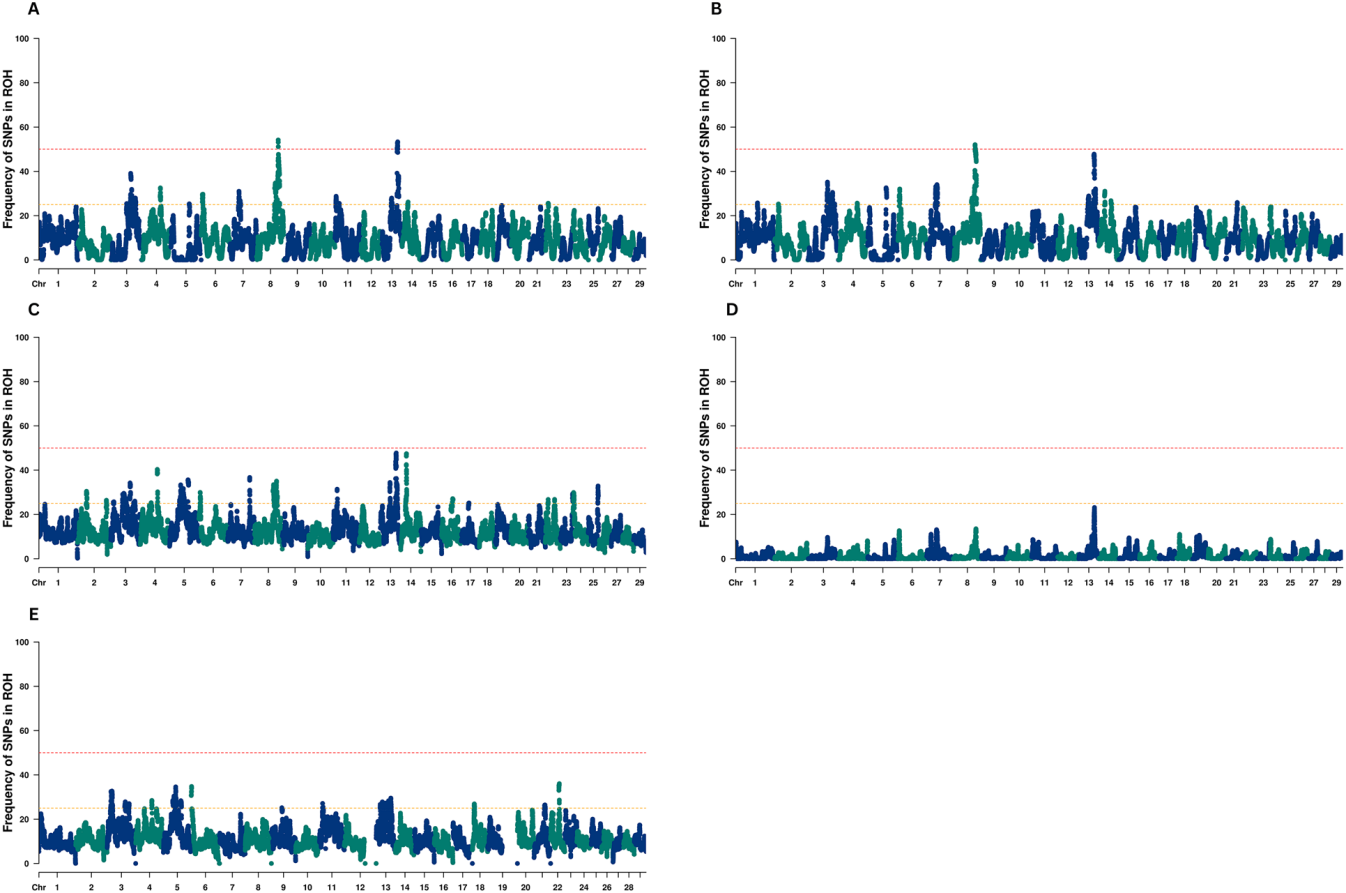
Frequency of markers within runs of homozygosity in American (**A**), Australian (**B**), Brazilian (**C**), Canadian (**D**), and Red Angus (**E**) cattle populations

Most ROHs in each population were between 2–4 Mb in length, ranging from 42% in BRA to 76% in CAA of all ROHs detected inside each population. Long ROHs (> 16 Mb) were observed in 0–3% of cases. Chromosomal coverage by ROH varied across populations, ranging from 3.02% on BTA1 in the RAA population to 11.8% on BTA28 in the AUS population. Figure 4 illustrates the distribution of SNPs located within ROH regions for each Angus population.

ROH islands were identified only in the AAA and AUS populations. In the AAA population, two ROH islands were identified: one located at BTA8:89,533,226–89,814,485 and another located at BTA13:62,469,591–65,307,514. These regions contain 61 markers, responsible for coding 100 genes and 418 QTLs associated with traits such as tenderness score, body weight, subcutaneous fat thickness, average daily gain, dry matter intake, and weight gain. In the AUS population, a single ROH island was identified at BTA8:89,533,226–91,302,171, containing 16 markers associated with 26 genes and 35 QTLs. These QTLs are associated with traits such as tenderness score, connective tissue amount, and marbling score. Emerging ROH islands may be forming in BRA on chromosomes BTA13 and BTA14.

### Generation proxy selection mapping

**Fig. 5:**
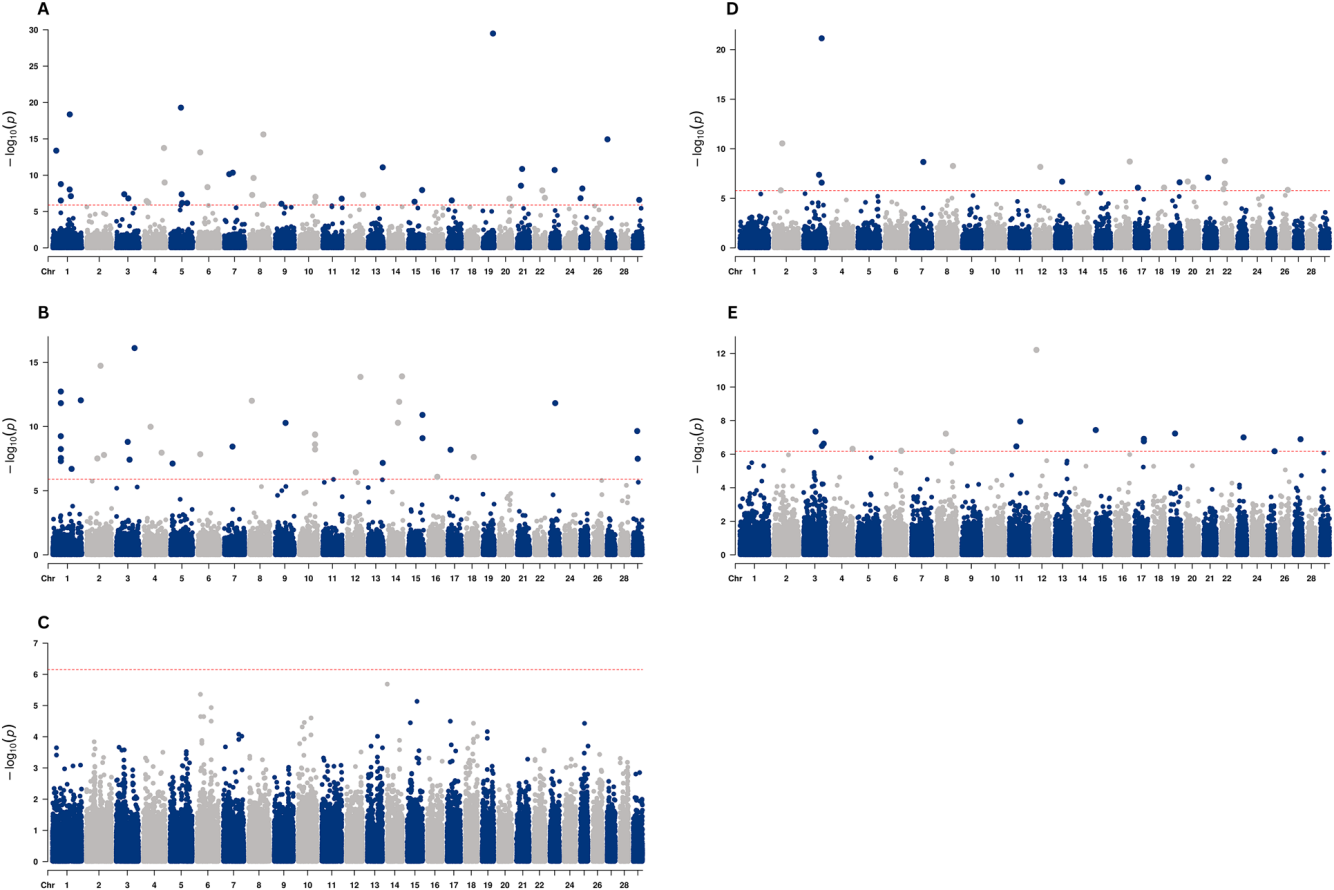
Generation proxy selection among American (**A**), Australian (**B**), Brazilian (**C**), Canadian (**D**), and Red Angus (**E**) cattle populations

**Fig. 6:**
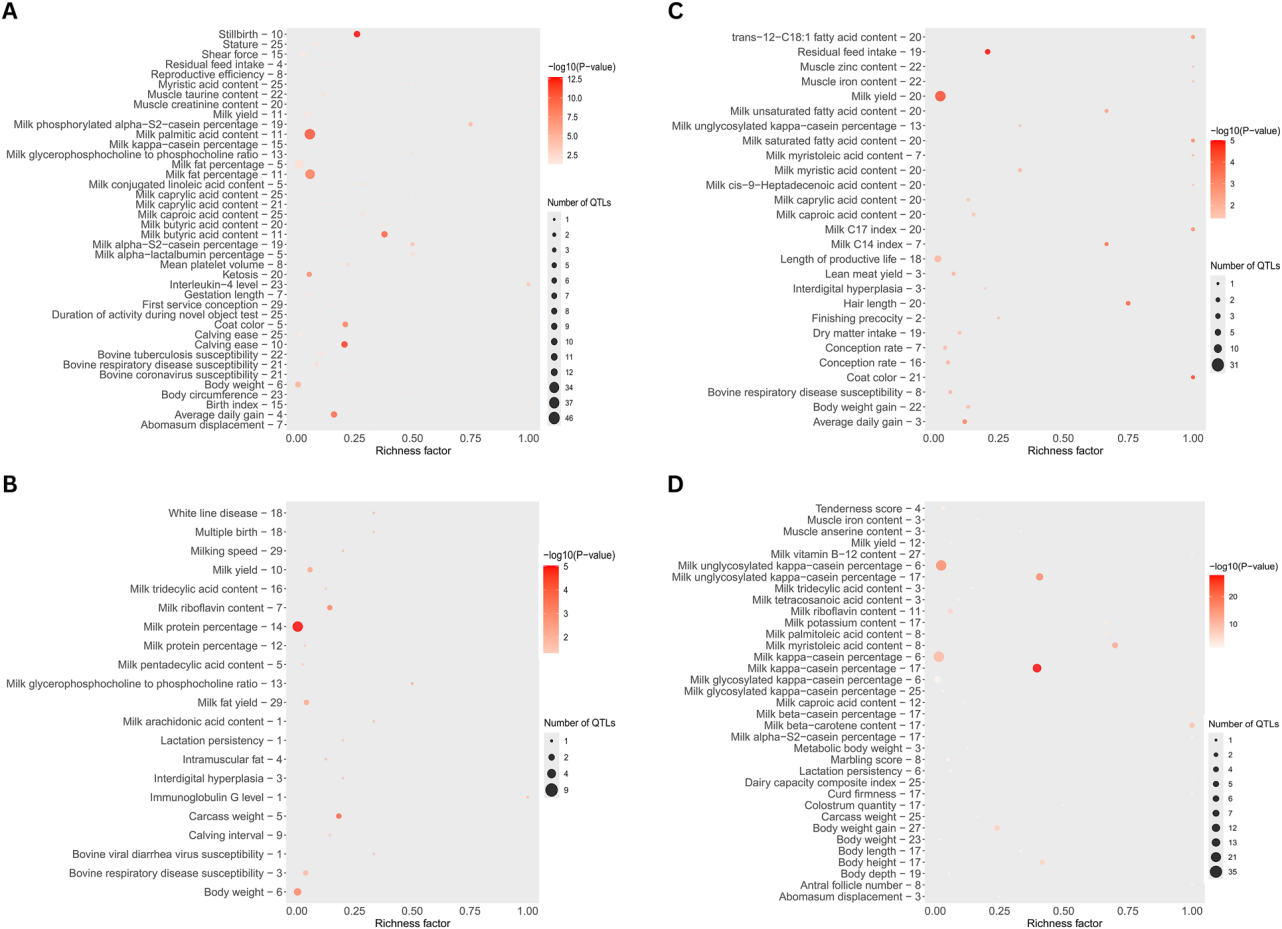
Enriched quantitative trait loci in the significant markers associated with the generation proxy selection in American (**A**), Australian (**B**), Canadian (**C**), and Red Angus (**D**) cattle populations

Figure 5 shows the GPSM results across all studied Angus populations, highlighting significant genomic regions under selection. Figure 6 shows the QTLs enriched for significant markers identified through the GPSM in the AAA, AUS, CAA, and RAAA populations. These enriched QTLs provide insights into the genetic basis of traits influenced by selection within each population. Interestingly, no significant markers were identified through the GPSM in the BRA population. In this population, the GPSM model explained only a small proportion of the variance in the generation proxy (PVE = 0.26), indicating limited temporal structure among genotyped animals. In contrast, the other populations presented moderate to high PVE values (RAAA = 0.42, AAA = 0.56, AUS = 0.59, and CAA = 0.76), confirming that most datasets contained sufficient temporal information for detecting allele frequency changes over time. These results suggest that the lower PVE observed for the BRA dataset likely limited the detection of significant selection signals in this population.

Populations AAA, AUS, CAA, and RAAA exhibited markers that have been subject to selection over time (Fig. 5). In the AAA population, 44 significant markers were identified, located within regions encoding 169 genes involved in 87 gene ontologies, and nearly 387 QTLs associated with traits such as body weight, body weight gain, average daily gain, metabolic body weight, tenderness score, marbling score, shear force, stature, residual feed intake, reproductive efficiency, coat color, and calving ease. In the AUS population, 39 significant markers were identified, located in regions encoding 105 genes and associated with 45 gene ontology terms. Furthermore, these markers are near 78 QTLs related to traits such as marbling score, intramuscular fat, body weight, body weight gain, tenderness score, carcass weight, and calving interval. In the CAA population, 20 significant markers were identified, located in regions encoding 77 genes, associated with amidase activity (GO:0004040) and fatty acid amide hydrolase activity (GO:0017064). The markers are located near 166 QTLs associated with traits such as dry matter intake, tenderness score, marbling score, body weight gain, body weight, hair length, residual feed intake, metabolic body weight, productive life span, lean meat yield, finishing precocity, conception rate, coat color, and average daily gain. In the RAAA population, 18 significant markers were identified, located within regions encoding 75 genes involved in 144 gene ontology terms related to biological processes, molecular functions, cellular components, and various metabolic pathways. Additionally, the markers are located close to 314 QTLs associated with traits like carcass weight, tenderness score, marbling score, body weight, body weight gain, metabolic body weight, carcass weight, body length, body height, and body depth. It is worth highlighting that, in our study, although each significant region encompasses several genes, the enrichment results provide insight into the biological processes most likely affected by selection, rather than pinpointing single causal loci.

## Discussion

One of the aims of this study was to explore the genetic similarities and differences among global Angus cattle populations. Figure 1 shows that the AAA, AUS, CAA, and BRA populations cluster closely together, indicating greater genetic similarity among these groups compared to the RAAA population. These findings suggest that, while all populations trace back to Scottish Angus origins (Vasconcellos et al. 2003; Nawaz et al. 2024), distinct selection practices over generations have contributed to the genetic distance between RAAA and the other populations. Regarding the proximity of the other populations, our results align with those reported by Carruthers et al. (2011), although their study used fewer markers and a smaller sample size (i.e., 190 animals with 22 microsatellite markers). Despite the influence of unique selection practices on each population, our findings suggest that gene flow through international genetic material exchanges (Cardoso et al. 2020; Angus Genetics Inc. 2023; ASBIA 2024) may help maintain similarities among these populations. This trend is evident across all population stratification results.

The small differentiation observed in the F_ST_ metrics (Table 3) for the AAA, CAA, AUS, and BRA populations also reflects their higher genetic similarity. The low F_ST_ values indicate minimal genetic differentiation between these breeds when compared pairwise with the RAAA population. According to Wright (1965), F_ST_ values between 0.05 and 0.15 suggest moderate differentiation, while values between 0.15 and 0.25 indicate high differentiation. These values can also be interpreted as a measure of genetic relatedness among populations (Hall 2022). Thus, individuals from the AAA, CAA, AUS, and BRA populations share a closer genetic relationship with each other than with individuals from the RAAA population. The moderate differentiation observed in the F_ST_ values between RAAA and the other populations suggests that the RAAA population has a distinct genetic profile. The level of differentiation observed between the Red Angus and other Angus populations (average F_ST_ ≈ 0.15) is comparable to that reported between distinct taurine breeds. For instance, previous studies found F_ST_ values of approximately 0.12 between Angus and Simental, 0.15 between Angus and Jersey (Kelleher et al. 2017), and higher than 0.20 between taurine and indicine breeds (Chen et al. 2023). This suggests that, despite sharing a common origin, the Red Angus population has diverged to an extent similar to that observed between recognized beef breeds, likely due to independent selection goals and restricted gene flow over time.

Combining genomic data from different populations can be a promising strategy to improve the accuracy of genetic predictions, particularly if the challenge of accounting for common ancestry between populations can be addressed (Cardoso et al. 2020). Integrating genomic evaluations has the potential to benefit beef cattle breeding programs by increasing the size of the training populations used in the genomic predictions (Alvarenga et al. 2023). However, this approach is only feasible and advantageous for all populations if the consistency of the gametic phase is similar across them. In other words, the persistence of LD between markers and QTLs must be comparable among the populations under study. The consistency of gametic phase represents the direction and strength of LD between pairs of loci (Brito et al. 2015), and its consistency across populations indicates that the association phase between markers and the underlying QTL alleles remains stable. Such stability ensures that genomic predictions based on SNP markers accurately capture the effects of causal variants in different populations. Our results show that the AAA, CAA, AUS, and BRA populations have high correlations, indicating that the phases of markers and QTLs are highly consistent across pairs of these populations. Similar findings have been reported for smaller subpopulations, such as AAA and AUS (Alvarenga et al. 2023), and BRA and CAA (Cardoso et al. 2020). Although the RAAA population had lower correlations with all other populations, it could still be incorporated into a combined evaluation. This would require increasing the number of common markers among populations to ensure consistent LD between markers and QTLs, thereby enabling more accurate predictions of breeding values. In other words, our results show that the high consistency of gametic phase among AAA, AUS, CAA, and BRA suggests that shared LD patterns would allow accurate joint genomic evaluations, supporting ongoing global evaluation initiatives. On the other hand, the lower consistency observed for RAAA indicates that this population would require increased marker overlap and reference population connectivity to ensure stable QTL-marker LD relationships.

The identification of ROHs, contiguous homozygous segments of the genome caused by shared ancestry, offers valuable insights into population history. The size of ROHs is inversely proportional to the number of generations since their formation. For instance, ROHs shorter than 2 Mb are estimated to have originated 25–50 generations ago, whereas ROH around 16 Mb in length likely originated about three generations ago (Tenhunen et al. 2024). The results shown in Table 4 and Fig. 3 suggest that many observed ROHs were formed in more distant generations. However, large ROH segments were also detected in almost every population studied. This is expected, as all the populations are under artificial selection, with only a subset of individuals in each population being used for breeding (Marras et al. 2015).

Despite the presence of ROHs, the concentration of ROHs in specific genomic regions, known as ROH islands, is particularly noteworthy. ROH islands can reflect the degree of genetic diversity within a population, as their distribution and pattern are population-specific (Mulim et al. 2022). As shown in Fig. 4, the associations have been effective in managing genetic diversity within populations. Interestingly, the number of ROH islands was small and detected in only two populations (i.e., AAA and AUS). Notably, the same ROH island was identified in both populations, while a second island on BTA13 nearly reached the threshold in the AUS population. These shared signatures of selection in AAA and AUS populations (i.e., on BTA8 for both populations and on BTA13 for AAA), highlight the influence of selection pressure related to economically significant traits such as tenderness score, body weight, subcutaneous fat thickness, average daily gain, dry matter intake, and weight gain. These traits have been prioritized by these breed associations and breeders (American Angus Association, 2024b; Byrne 2015). These findings are particularly relevant for breeding strategies, as ROH islands highlight genomic regions that have been consistently targeted by selection for economically important traits. Understanding the distribution and overlap of ROH islands across populations helps identify loci that contribute to desirable phenotypes while revealing areas where genetic diversity should be preserved. Thus, ROH-based insights can guide the design of balanced selection programs that maintain genetic variability without compromising productivity.

It is important to note that the overlap of significant genomic regions with QTLs associated with multiple trait categories may reflect either true pleiotropic effects or, alternatively, the clustering of adjacent QTLs due to limited mapping resolution and annotation density in the Animal QTLdb. Many genomic regions under selection, such as those on BTA8 and BTA13, include genes involved in metabolic and growth-related pathways that could influence multiple correlated phenotypes. Similar observations have been reported in recent studies exploring pleiotropy and shared selection signatures in cattle (Kemper and Goddard 2012; Kemper et al. 2015; Fathoni et al. 2025). Although the present data do not allow us to disentangle pleiotropy from linkage, the functional convergence of these regions suggests that selection may be acting on genomic segments with broad phenotypic effects.

Within these ROH islands, key genes such as *CEP250* (Centrosomal Protein 250) and *ERGIC3* (ERGIC and Golgi 3) were identified, both of which have been associated with growth and meat quality in beef cattle (Mudadu et al. 2016). Additionally, genes including *C13H20orf173* (chromosome 13 C20orf173 homolog), *SPAG4* (Sperm Associated Antigen 4), *NFS1* (NFS1 Cysteine Desulfurase), *CPNE1* (Copine 1), and *ROMO1* (Reactive Oxygen Species Modulator 1) have been previously reported in American and Australian Angus subpopulations as being associated with foot angle (Alvarenga et al. 2023). Interestingly, the same genomic region has also been identified as a selection signature in Angus cattle from Russia (Kolpakov et al. 2024). These findings underscore the influence of selection pressure on specific genomic regions across populations and emphasize the importance of traits such as growth, meat quality, and structural soundness in breeding objectives. The shared ROH islands between the AAA and AUS populations, along with their overlap with regions reported in other studies, highlight the consistent selection for economically important traits within Angus cattle globally. These results offer valuable insights into the genetic mechanisms driving breed improvement and provide a foundation for further exploration of selection signatures and their functional impacts on performance traits. In the CAA population, a lower number of ROHs was observed compared to the other groups. To further investigate this, we performed an additional analysis using more flexible detection parameters. This approach resulted in the identification of a higher number of short ROHs; however, no substantial clustering of ROHs at specific genomic regions was detected. The results of this complementary analysis are provided in Supplementary Material 4.

The GPSM method is an effective approach for identifying loci under selection in livestock populations, offering accurate detection of selected loci and insights into shared genetic architectures across breeds and populations (Rowan et al. 2021; Grohmann et al. 2023). As highlighted in the GPSM results section, markers near QTLs associated with body weight, maternal traits, growth, and meat quality have shown frequency changes in the AAA, AUS, CAA, and RAAA populations. Interestingly, these traits are explicitly considered in the evaluations performed by each association (American Angus Association,^2024^b; Byrne 2015; Canadian Angus Association, 2024b; Red Angus Association of America, 2024). This finding demonstrates that selection efforts by the associations have successfully altered the frequencies of markers and genes linked to these economically important traits. Moreover, it underscores how selection strategies, tailored to specific breeding objectives, have effectively driven genetic changes aligned with industry priorities.

Interestingly, a partial overlap between ROH islands and GPSM-identified selection regions in AAA and AUS, particularly on BTA8, where long-term homozygosity coincides with ongoing allele frequency change, was observed. This suggests that these regions were originally shaped by historical selection and continue to respond to more recent directional selection for carcass and growth traits. In contrast, populations without ROH islands but with significant GPSM signals (CAA and RAAA) show evidence of more recent or polygenic selection spread across the genome.

## Conclusions

Our findings provide valuable insights into the genetic architecture and selection history of Angus cattle populations raised worldwide. The high degree of genetic similarity observed among the AAA, AUS, CAA, and BRA populations underscores the influence of shared selection goals and international genetic exchanges in maintaining breed uniformity. In contrast, the distinct profile of the RAAA population reflects the impact of divergent selection practices. The identification of ROH islands and selection signatures linked to economically significant traits further highlights the success of targeted breeding programs in shaping the genomic landscape of these populations. These results not only evidence the current selection strategies used by breed associations but also offer a foundation for enhancing genetic evaluations. Future studies should focus on integrating these findings to identify novel traits, optimize selection methodologies, and refine genomic predictions, ultimately driving the advancement of sustainable and efficient beef production systems worldwide.

## Supplementary Information

Below is the link to the electronic supplementary material.

Supplementary material 1 (XLSX 16.5 kb)

Supplementary material 2 (XLSX 24.8 kb)

Supplementary material 3 (PDF 13.3 kb)

Supplementary material 4 (PDF 8.3 kb)

Supplementary material 5 ( 9464.4 kb)

## Data Availability

The datasets for this article are not publicly available because they are the property of the American Angus Association, Angus Australia, Canadian Angus Association, PROMEBO Beef Cattle Breeding Program producers and Embrapa, and Red Angus Association of American and this information is commercially sensitive. For scientific research purposes, the data requests should be forwarded along with the research proposal to KR (kretallick@angus.org), Carel Teseling (carel.teseling@angusaustralia.com.au), KL (klatimer@cdnangus.ca), FFC (fernando.cardoso@embrapa.br), and AJK (aj@redangus.org), respectively.
