# Supplementary figures and images for: Genomic structure and selection history across Angus populations worldwide: insights from ROH, selection mapping, and functional analyses
Source: Mamm Genome. 2025 Dec 22;37(1):19. doi: 10.1007/s00335-025-10188-y (PMC12722264; doi:10.1007/s00335-025-10188-y)

American Angus

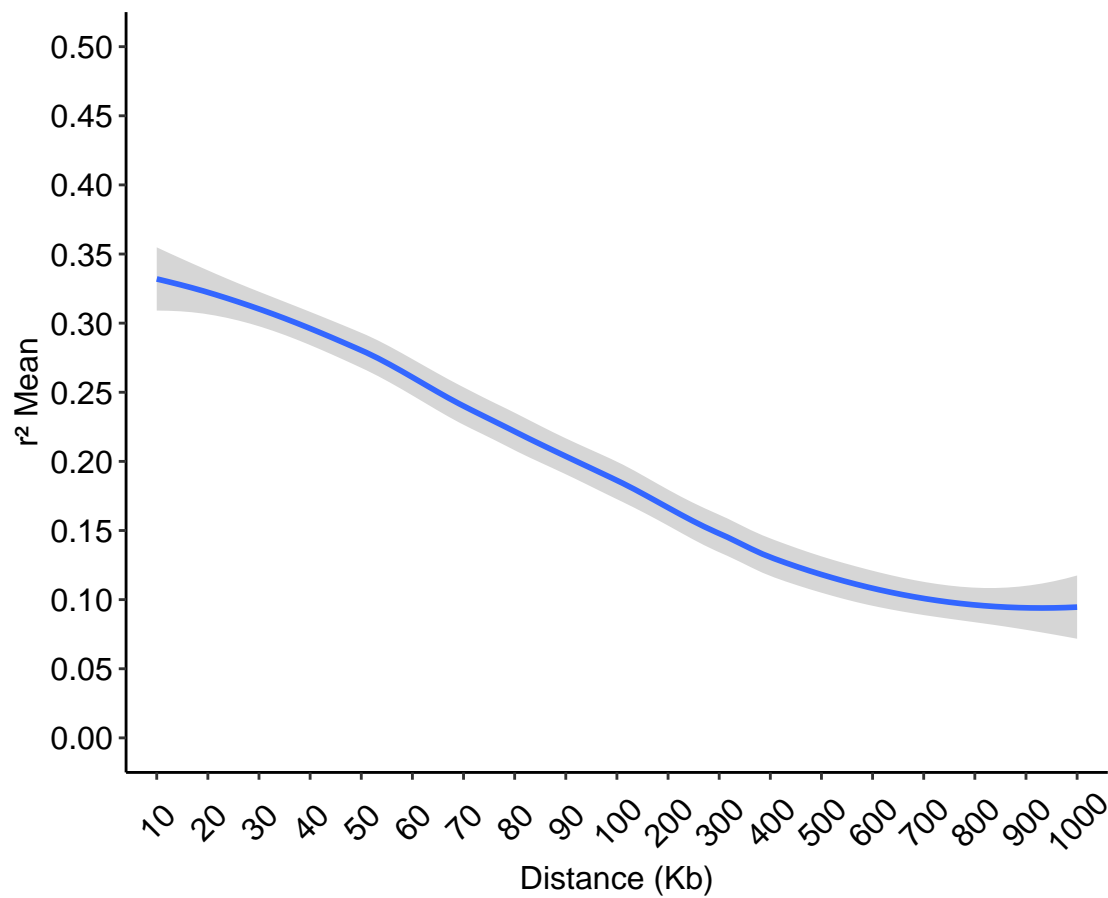

Canadian Angus

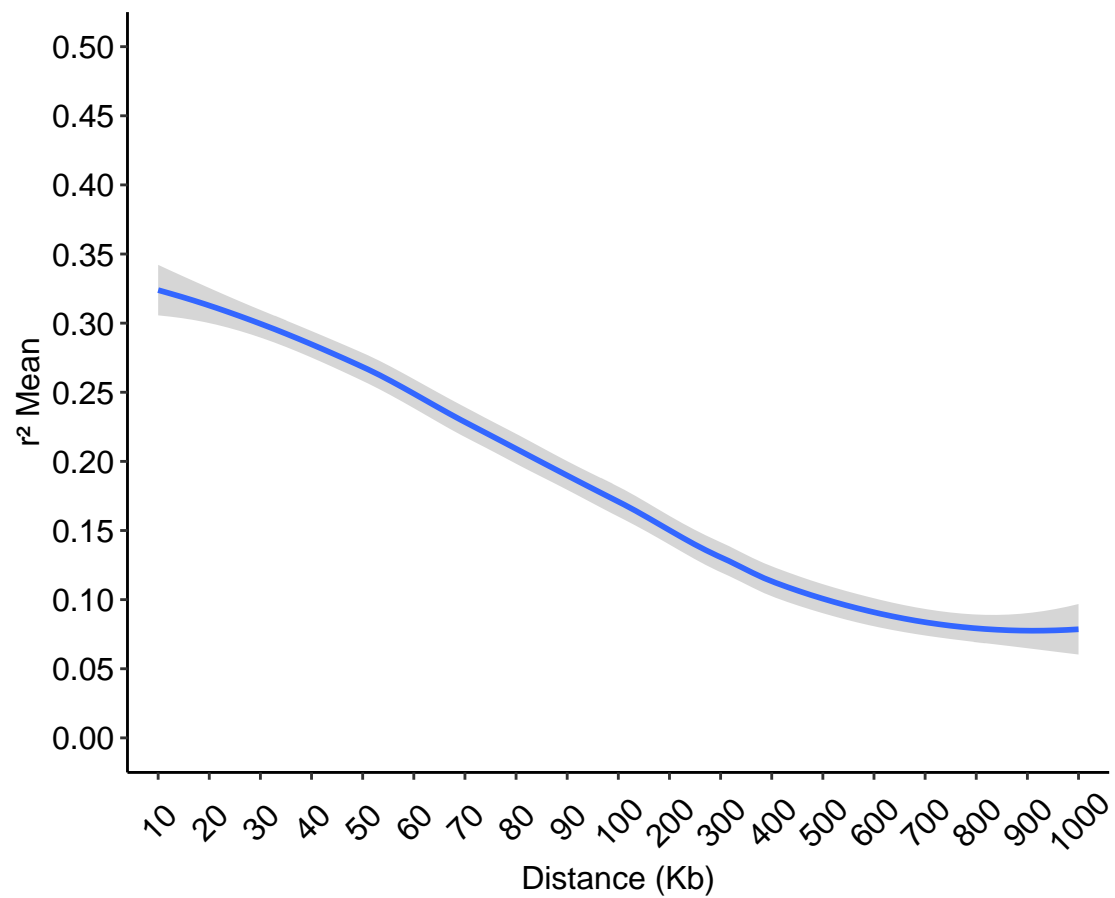

Australian Angus

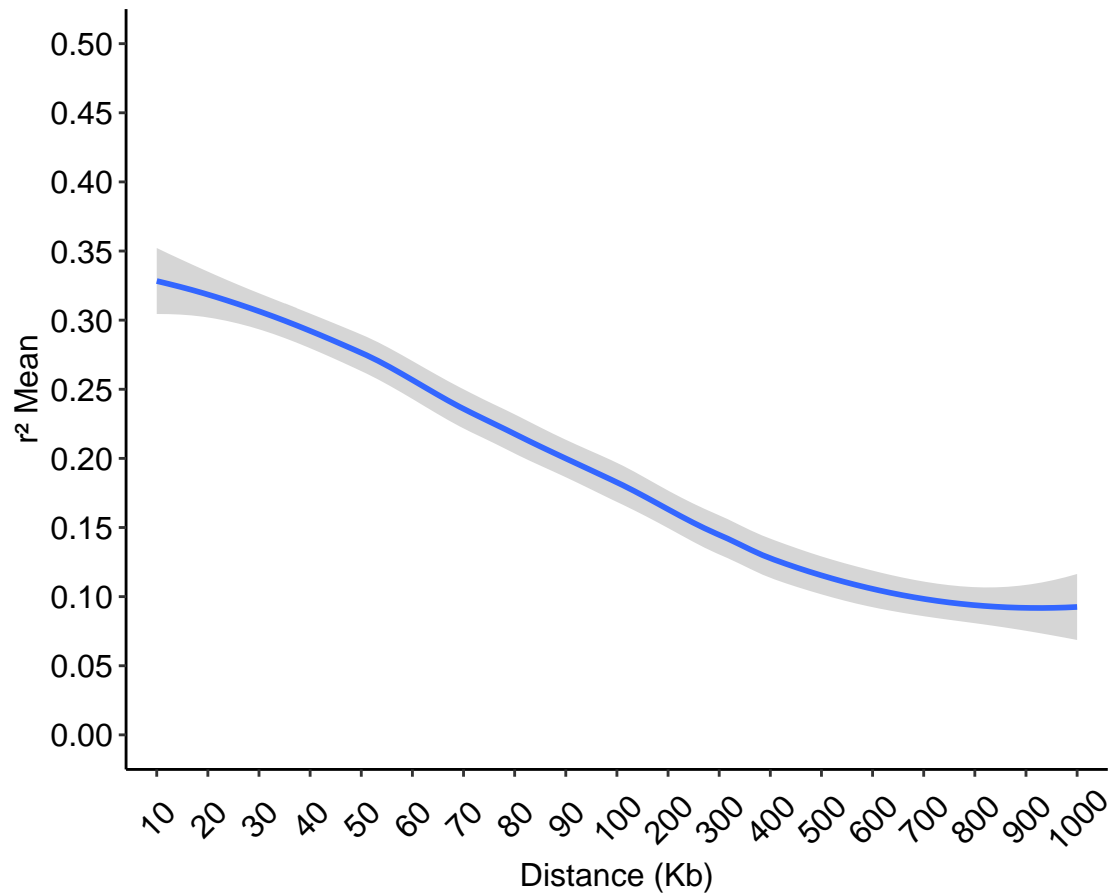

Brazilian Angus

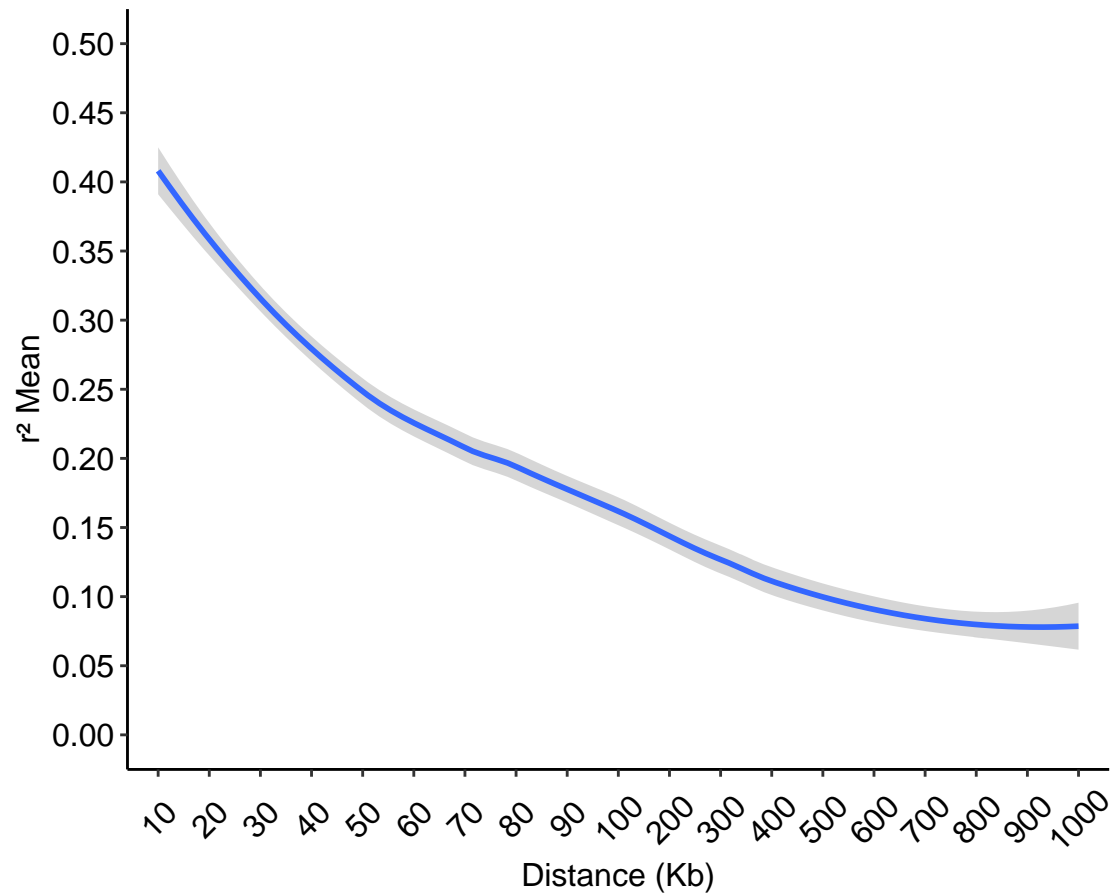

Red Angus

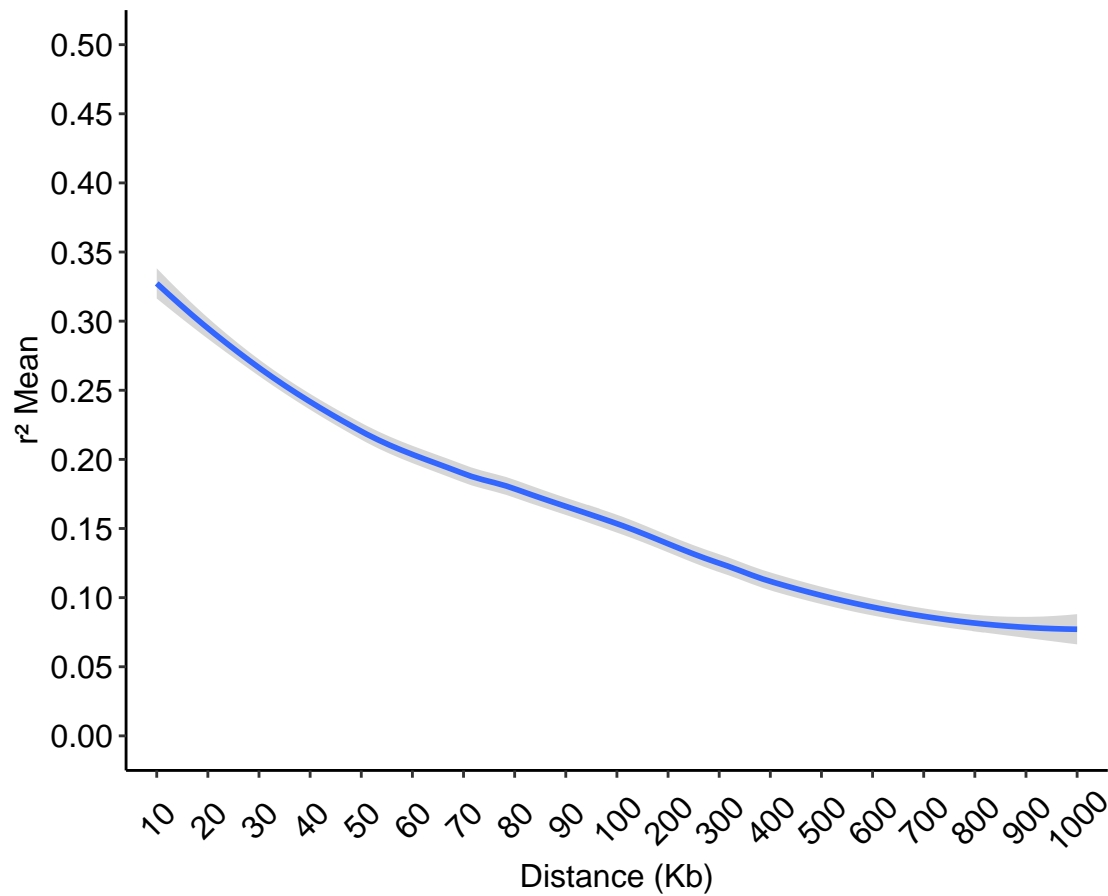

Supplement: Supplementary file 3 — Supplementary material 3 (PDF 13.3 kb) [file 335_2025_10188_MOESM3_ESM.pdf]

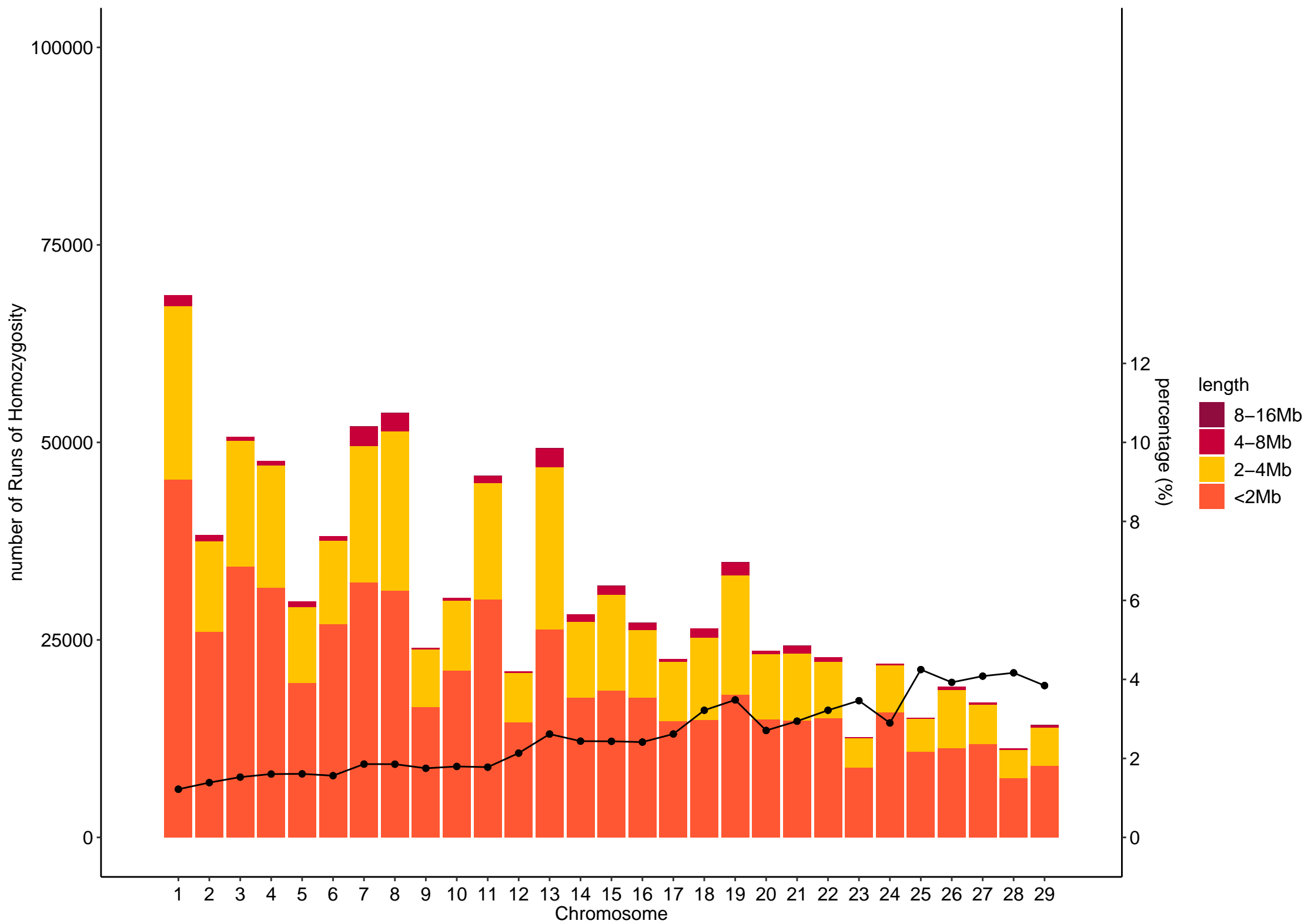

Supplement: Supplementary file 4 — Supplementary material 4 (PDF 8.3 kb) [file 335_2025_10188_MOESM4_ESM.pdf]
